# Multiparametric and semiquantitative scoring systems for the evaluation of mouse model histopathology - a systematic review

**DOI:** 10.1186/1746-6148-9-123

**Published:** 2013-06-21

**Authors:** Robert Klopfleisch

**Affiliations:** 1 Department of Veterinary Pathology, College of Veterinary Medicine, Freie Universität Berlin, Berlin, Germany

**Keywords:** Score, Semiquantitative, Histopathology, Colitis, Nephritis, Hepatitis, Encephalitis

## Abstract

**Background:**

Histopathology has initially been and is still used to diagnose infectious, degenerative or neoplastic diseases in humans or animals. In addition to qualitative diagnoses semiquantitative scoring of a lesion`s magnitude on an ordinal scale is a commonly demanded task for histopathologists. Multiparametric, semiquantitative scoring systems for mouse models histopathology are a common approach to handle these questions and to include histopathologic information in biomedical research.

**Results:**

Inclusion criteria for scoring systems were a first description of a multiparametric, semiquantiative scoring systems which comprehensibly describe an approach to evaluate morphologic lesion. A comprehensive literature search using these criteria identified 153 originally designed semiquantitative scoring systems for the analysis of morphologic changes in mouse models covering almost all organs systems and a wide variety of disease models. Of these, colitis, experimental autoimmune encephalitis, lupus nephritis and collagen induced osteoarthritis colitis were the disease models with the largest number of different scoring systems. Closer analysis of the identified scoring systems revealed a lack of a rationale for the selection of the scoring parameters or a correlation between scoring parameter value and the magnitude of the clinical symptoms in most studies.

**Conclusion:**

Although a decision for a particular scoring system is clearly dependent on the respective scientific question this review gives an overview on currently available systems and may therefore allow for a better choice for the respective project.

## Background

Histopathology has initially been and is still used today to diagnose infectious, degenerative or neoplastic diseases in humans or animals. These qualitative diagnoses are based on a sum of observable changes in the morphology of the analyzed tissue. The cognition of these changes is based on the pattern recognition of the observer and the comparison of these patterns with the known physiologic variation in tissue morphology in the respective species. Decades of experience in veterinary pathology show that this approach allows for reproducible qualitative diagnoses by the observer but can also be used for semiquantitative scoring of the lesions magnitude, i.e. on an ordinal scale for instance with a low, medium or high grade trichotomy which correlates with the clinical relevance of the lesions.

Absolute quantification of the lesions extent and severity is however difficult since two main problems hamper absolute quantification, i.e. on a rational scale with absolute values of 1, 2, 3 etc., using standard, non-automated histopathology. First, the detection method is not reliable enough. Despite intensive training and attempts to standardize nomenclature and the definition of lesions there are still unresolved issues in terms of interobserver variation which may be acceptable for qualitative and semiquantitative evaluation but not for absolute quantitation [1]. Second, in most circumstances it is impossible to objectively justify the interval between two values, thus a read out of histopathologic scoring on a rational scale is impossible.

Image analysis by automated calculation of the tissue area affected or cells present per area have been introduced to overcome this problem. These approaches aim at a reliable and reproducible histopathology read out in a rational scale to allow proper statistical processing and at an exclusion of an observer bias [2]. Image analysis approaches usually use one or only few two dimensional planar sections of the tissue of interest to measure three-dimensional objects. This two-dimensional approach thus may also lead to biased results. Stereology, which is based on systematic random sampling and estimates third dimensional information, has been developed to avoid this bias [3]. It can therefore be seen as the most sophisticated method for the quantification of histologic information. It is however by comparison a laborious and complex method which is established in only few laboratories.

Semiquantitative scoring systems are therefore still the most widely used methods to include histopathologic information in biomedical research. These scoring systems usually include multiple parameters which are separately quantified on an ordinal scale and finally combined in a total score. Average scores of the different experimental groups can then be compared by non-parametric statistical tests. The selection of the parameters should be based on the scientific hypothesis or question together with the current knowledge on the morphologic outcome of the investigated disease model. It may therefore be useful to design individual scoring system for each study which in the best possible way answers the particular scientific question. Standard scoring systems for specific disease models on the other hand allow for the comparison of the results of different studies.

Several standard scoring systems for different mouse models have been introduced or emerged in the past 20 years. Histopathologists are therefore repeatedly requested by cooperating scientists to evaluate the outcome of animal studies using standard scoring systems or to elaborate project specific scoring systems. The present review is intended to give a comprehensive overview on the currently most commonly used multiparametric, semiquantitative scoring systems for mouse model histopathology.

## Results

### Scoring systems for murine intestinal disease models

**Figure 1:**
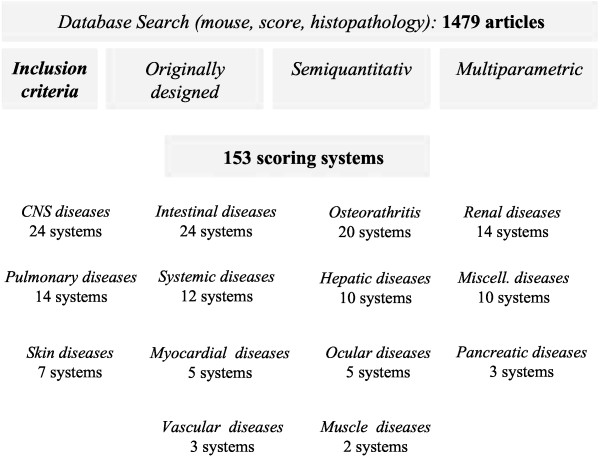
Flow chart visualizing the approach to identify 153 original, multiparametric, semiquantiative scoring systems by Pubmed data search.

Eighteen original scoring systems for colitis models could be identified. Most of these scoring systems were designed for dextran sodium sulfate (DSS)-induced colitis models but 2,4,6-trinitrobenzene sulfonic acid (TNBS)-induced colitis and several models of immunopathologic colitis were also used to establish scoring systems (Table 1, Figure 1). Eight scoring systems for DSS-induced colitis fulfilled all required parameters and were included in this review. Generally, all of the paper with original DSS colitis scoring systems had a high citation rate but the scoring system described by Cooper is one of the earliest system with the highest citation number up to date and can therefore be seen as a prototype for DSS-colitis scoring [4]. It separates the colon into three segments which are then scored by the parameters of crypt loss, inflammation and affected area. Although later studies refined and increased the number of histopathologic parameters the value of this initial study is the separation of the colon in three segments and the sophisticated approach to the establishment of the scoring system. Remarkably, parameters in this study were chosen and tested according to their correlation with the clinical symptoms of the mice. This is contrast to the vast majority of scoring systems presented in this review, which only rarely stated the rational for choosing the included parameters and did not perform a correlation with the clinical symptoms.

**Table 1:** Semiquantitative scoring systems for murine intestinal disease models

**Disease model**	**Scoring system: parameters (scale width)**	**Citations**
***Colitis***		
DSS-induced [4]	Proximal + middle + distal colon: crypt loss (0–4), inflammation (0–3) both quantified by the affected area (0–4)	718
DSS-induced [5]	Extent, inflammation, necrosis, regeneration (each 3–0)	321
DSS-induced [6]	Crypt damage, area involved, regeneration (each 0–4), extent (0–3), inflammation (0–3),	255
DSS-induced [7]	Crypt loss (0–4), crypt distortion (0–4), epithelial hyperplasia (0–4), inflammation (0–3) each multiplied by percentage of involved area (0–4)	185
DSS-induced [8]	Hyperplasia (0–3), severity (0–3), ulceration (0,1), area involved (0–4)	147
DSS-induced [9]	Severity of inflammation, thickness of inflammation, epithelial damage character, extent of epithelial damage (each 0–3)	130
DSS-induced [10]	Epithelium (goblet cell/crypt loss), infiltration (each 0–4)	103
DSS-induced [11]	Infiltration, tissue damage (each 0–3)	89
TNBS-induced [12]	Inflammation, loss of goblet cells, vascular density, transmural infiltration, thickening of the colon wall (together 0–4)	788
TNBS-induced [13]	Crypt distortion, goblet cell loss, acute inflammation, chronic inflammation (each 0–2)	126
TNBS-induced [14]	Loss of mucosal architecture, cellular infiltration, muscle thickening (each 0–3), crypt abscesses (0,1), goblet cell depletion (0,1)	97
Acetic acid-induced [15]	Inflammation, bleeding, ulcer size, deepness, perforation (together 0–6)	132
TNBS-induced [16]	Percentage of area, crypt loss (both 0–4) number of follicles, edema, erosion/ulceration, infiltration (each 0–3)	57
HLA-B27 transgenic mice [17]	Inflammation, goblet cell loss, mucosal thickening, submucosal infiltration, architecture loss (each 0–4), ulcer, crypt abscess (each 0,1)	423
IL10-deficient mice [18]	Mucosal ulceration, epithelial hyperplasia (both 0–3), Lamina propria mononuclear infiltrate, Lamina propria neutrophil infiltrate (each 0–2)	423
IL10-deficient mice [19]	Inflammation/epithelial erosion/ulcers/hyperplasia/crypt abscesses/goblet cell depletion (together 0–4) multiplied by no. of affected colon segments (1–5)	555
MHC missmatch [20]	Active inflammation (0.5-3), chronic inflammation (0.5-3), villous architecture (1–3) multiplied by the affected area (0.5-4)	83
Amebic colitis [21]	Number of amoeba (0–5), ulceration (0-100%), inflammation (0–5)	58
***Small intestinal diseases***		
Clostridial toxicosis [22]	Epithelial damage, hyperemia/edema, neutrophils (each 0–3)	202
Intestinal ischemia [23]	Normal mucosa (0), villous edema (1), subepithelial edema (2), epithelium loss at villi sides (3), denuded villi (4), loss of villous tissue (5), crypt infarction (6), transmucosal infarct (7), transmural infarct (8)	270
Intestinal ischemia [24]	Mucosal damage, inflammation, hyperemia/hemorrhage (each 0–5)	72
Jejunitis [25]	Villous length, villous tips, epithelium, inflammation, crypt loss (each 0–2), crypt abscesses, hemorrhage (each 0,1)	15
Bacterial ileitis [26]	Hemorrhage, villous atrophy/necrosis, edema, congestion, neutrophils, epithelial necrosis (each 0–3)	1
***Gastric diseases***		
*Helicobacter*-induced [27]	Five areas multiplied by inflammation (0–3)	49

*DSS*, Dextran sodium sulfate; *TNBS*, 2,4,6-trinitrobenzene sulfonic acid, *MHC*, Major histocompatibility complex; *HLA-B27*, Human Leukocyte Antigen-B27, *IL10*, Interleukin 10.

When comparing all original colitis models it becomes obvious that a wide variety of appellation for the most common parameters inflammation, crypt and surface epithelial damage were used (Table 1). These differences in the nomenclature make it however difficult to directly compare the different scoring systems. Less often used parameters in the colitis scoring systems were goblet cell loss, regeneration, muscular and epithelial hyperplasia, edema and the separation between acute and chronic inflammation. In some cases these singularities of the respective scoring system seem to be dependent on the objectives of the study while in most cases the rationale for selection of the parameters was not given.

The number of identified scoring systems for small intestinal disease was significantly lower than for colitis models and had on average lesser citations (Table 1). Two independent scoring systems were identified for intestinal ischemia which include the comprehensible parameters of hyperemia and hemorrhage as well as inflammation and epithelial damage and in the case of the higher cited publication by Park et al. several other more sophisticated parameters [23,24]. Only two scoring systems for small intestinal enteritis were detected which both included villous morphology, epithelial damage and inflammation as the main features (Table 1). Surprisingly, only one semiquantitative scoring system for gastritis was identified. Wang et al. scored the severity of *Helicobacter*-induced gastritis in a uniparametric scoring of five gastric areas. For the sake of completeness this studies was included in this review although it did not fulfill the criterion of multiple parameters [27].

### Scoring systems for murine osteoarthritis models

Seventeen original semiquantitative, multiparametric scoring systems were identified for murine osteoarthritis models (Table 2). Three of these, designated as osteoarthritis in Table 2, are scoring systems for human idiopathic arthritis and were transferred to the murine model to allow for comparisons of the model with the human disease. Of these the the score developed by Mankin et al. has by far the highest citation number which is most probably influenced by its common application in the description of human osteoarthritic lesion [28]. The majority of mouse specific systems were established in collagen-induced models while models of instability-induced or bacterial arthritis models were only rarely used. Of these, the scoring system by William et al. had the highest citation number [29]. Twelve of the thirteen osteoarthritis scoring systems include an evaluation of cartilage damage, nine included an infiltration parameter, seven included changes in perichondral bone structure and six studies evaluated the extend of synovial infiltration (Table 2). Other parameters like tidemark integrity, proteoglycan content of the cartilage, pannus formation or synovial hyperplasia were used only in the minority or single scoring systems. Again, in most of these descriptions of original scoring systems no rational for inclusion of the respective parameters is given but their usefulness can be comprehended by reflecting the general pathogenesis of osteoarthritis.

**Table 2:** Semiquantitative scoring systems for murine osteoarthritis models

**Osteoarthritis model**	**Scoring system: parameters (scale width)**	**Citations**
Arthritis [28]	Cartilage structure (0–6), cells (0–3), Safranin-O-stain (0–4), tidemark integrity (0–1)	1322
Arthritis [30]	Cartilage destruction (0–6), optional subgrading (subdivision in 2 subgrades)	197
Arthritis [31]	Synovial lining, resident cell density, inflammation (each 0–3)	49
Collagen-induced [29]	Extent of synovitis, cartilage loss, bone erosions (together 0–3)	713
Collagen-induced [32]	Inflammation, cartilage destruction, bone erosion (each 0–3)	273
Collagen-induced [33]	Infiltration in the exudate, infiltration of the synovial membrane, cartilage destruction, bone erosion (each 0–3)	198
Collagen-induced [34]	Joint exudate (0–5), proteoglycan depletion (0–3)	139
Collagen-induced [35]	Bone resorption, inflammation, cartilage damage (each 0–5)	95
*Mycobacterium butyricum*-induced [36]	Synovial thickening, infiltration, pannus formation (each 0–3)	95
Adjuvant-induced [37]	Percentage of affected area, synovial hyperplasia, cartilage destruction, bone erosion (each 0–3)	64
IL1-induced arthritis [38]	Synovial infiltration, proteoglycan depletion, cartilage damage (each 0–3)	41
Instability-induced [39]	Matrix structure, matrix staining, cellularity, subchondral bone (each 0–8)	85
Instability-induced [40]	Cartilage destruction (0–4), osteophyte formation (0–3)	85
Hereditary arthritis [41]	Articular cartilage structure (0–8), Toluidine blue-staining (0–6)	52
Bacterial arthritis [42]	Infiltration (0–4), pannus formation, cartilage destruction, extra-articular manifestations, tail lesion (each 0,1)	145
Cartilage repair [43]	Cell morphology (0–4), matrix staining (0–3), surface regularity (0–3), thickness (0–2), integration of donor cartilage (0–2)	819
Cartilage repair [44]	Cellular morphology (0,2,4), Safranin-O stain (0–3), structural regularity (0–3), structural integrity (0–2), thickness (0–2), bonding (0–2), hypocellularity (0–3), chondrocyte clustering (0–2), adjacent degeneration (0–3)	297
Cartilage repair [45]	Relative defect area (0–4), integration of repair tissue (0–3), matrix staining (0–4), cellular morphology (0–5), defect architecture (0–4), surface architecture, percentage of new bone (0–4), tidemark formation (0–4)	222
Cartilage repair [46]	Filled depth (0–4), integration (0–2), surface architecture (0–3), cell morphology (0–3), cellularity (0–2), tidemark formation (0–4), Toloudin blue stain (0–2)	130
Cartilage repair [47]	Defect filling (0–4), osteochondral reconstruction (0–2), matrix staining (0–4), cell morphology (0–4)	96

*IL1*, Interleukin 1.

### Scoring systems for murine renal disease models

Fourteen original multiparametric, semiquantitative scoring systems for murine models of renal diseases fulfilled the required criteria for inclusion in this review (Table 3). Scoring systems for murine *Lupus erythematous* models were the dominant model in the category of renal disease models with four appearances. Austin et al. published the lupus nephritis score with the highest citation number [48]. It uses a complex scoring system with 10 parameters and a scale width of four and five respectively and was thus more sophisticated than the other scoring systems which used only four different parameters.

**Table 3:** Semiquantitative scoring systems for murine renal disease models

**Renal disease model**	**Scoring system: parameters (scale width)**	**Citation**
Lupus nephritis [48]	Activity index (glomerular/tubulointerstitial abnormalities (6-tier, each 0–4)); chronicity index (4-tier, each 0–3)	358
Lupus nephritis [49]	Mesangial thickening, extent of changes (together 0–4)	44
Lupus nephritis [50]	Glomerular cell proliferation, lobulation, hyaline droplets, macrophage infiltration (together 0–3)	25
Lupus nephritis [51]	Glomerular inflammation, proliferation, crescent formation, necrosis (each 0–3)	1
Toxic nephropathy [52]	Glomerular cellularity, hypertrophy, thrombosis, dilation (together 0–5)	4
Toxic nephropathy [53]	Glomerular injury, tubular cysts/casts, podocyte hyperplasia, interstitial inflammation (each 0–4)	33
Hypertension nephropathy [54]	Mesangial matrix, percentage of glomerular affection (each 0–4)	524
Diabetic-/Hypertension-induced glomerulosclerosis [55]	Arteriole hyalinization, glomerular sclerosis (0–4), interstitial volume (%)	152
Diabetic nephropathy [56]	Glomerulosclerosis, interstitial fibrosis (each 0–4)	15
Crescentic glomerulonephritis[57]	Fibrin deposition, immunoglobulin deposition, tubular damage, glomerular crescents (each 0–3)	8
MRL/MPJ mouse glomerulonephritis [58]	Glomerular infiltration, crescents, necrosis, tubular casts, interstitial infiltrates (each 0–4)	244
Age-associated changes [59]	Mesangial proliferation, sclerosis, hyalinization (together 0–5)	27
HIV-nephropathy [60]	Tubuloepithelial degeneration/regeneration, tubular casts, dilation, interstitial infiltration, glomerular sclerosis, collapse, podocyte hyperplasia (each 0–3)	47
Obstructive nephropathy [61]	Tubular dilation/atrophy, interstitial fibrosis (each 0–3)	4

*HIV*, Human immunodeficiency virus; *MRL*, Murphy Roths Large.

Glomerular cellularity and proliferation were the terms most commonly used in all renal scoring systems except one scoring system for obstructive nephropathy model [61]. In addition, one half of the systems included tubulointerstitial infiltration and fibrosis in the scoring system.

### Scoring systems for murine models of neurologic disease

Twenty-two original scoring systems for murine models of central nervous system (CNS) disease were identified (Table 4). Scoring systems for experimental autoimmune encephalomyelitis (EAE) and stroke clearly dominated results. Due to the wide variety of diseases covered by the system the selection of parameters to be analyzed also had a wide variation and was clearly dependent on the pathophysiology of the disease. But again, the rationale for inclusion of parameters was not consistently given.

**Table 4:** Semiquantitative scoring systems for murine central nervous disease models

**Disease model**	**Scoring system: parameters (scale width)**	**Citations**
***Stroke***		
Focal ischemia [62]	Ischemic neuronal damage (0–3)	2176
Focal Ischemia[63]	18 areas x neuronal injury (0–5)	23
Global ischemia [64]	Infarcts in 3 cerebral regions (0–4), hippocampus infarction (0–4)	100
Global ischemia [65]	Eight regions x neuronal cell los/gliosis/iron deposition/gliosis (0–3)	95
Peripheral nerve ischemia [66]	Edema, fiber regeneration (each 0–4)	21
*Multiple sclerosis models*		
EAE [67]	Inflammation, neuronal degeneration (each 0–4)	160
EAE [68]	Inflammation (cells/cuff), axonal injury, axonal loss (each 0–4)	81
EAE[69]	Lesion severity, myelin loss/tissue injury, acute inflammation, chronic inflammation (each 0–5)	79
Theiler's murine encephalomyelitis virus [70]	Neuropil inflammation, demyelination, necrosis, meningeal inflammation (each 0–4)	77
EAE [71]	Infiltration, demyelination (together 0,1) in 16 regions	64
EAE [72]	Inflammation, necrosis (each 0–3)	53
EAE [73]	Inflammation (cuffs/100 m^2^), demyelination (lesions/mm^2^)	52
EAE [74]	Meningitis (0–2), perivascular cuffing (0–5), demyelination (0–3)	43
EAE [75]	Spinal cord demyelination (0–3), inflammatory cells (No./mm^2^)	22
***Spinal cord trauma (SCT)***		
SCT [76]	Gray matter degeneration/infarction (0–4) in 5 μm serial sections	33
SCT [77]	150 μm intervals x Area affected by neuronal degeneration, malacia (0–4)	23
*Encephalitis/Meningitis*		
Streptococcus meningitis [78]	Meningeal inflammation (0–3) x four regions	46
*Trypanosoma encephalitis *[79]	Meningitis, perivascular cuffing, neuropil infiltration (each 0–4)	41
***Miscellaneous CNS diseases***		
Oxidative damage [80]	7 areas x necrosis (0–3)	27
Senescence [81]	Spongiosis (0–3), lipofuscin positive cells (%)	23
Spinocerebellar ataxia [82]	Molecular layer thickness, Purkinje cell loss (each 0–3)	10

*EAE*, Experimental autoimmune encephalomyelitis, *SCT*, Spinal cord trauma, *CNS*, Central nervous system.

A striking feature of CNS disease scoring systems was the relatively low number of ordinal scales for parameter´s magnitudes and the common inclusion of multiple anatomical sites into the scoring system (Table 4). This discrepancy is not addressed in the respective publications but may be based on the anatomical diversity of the CNS. Furthermore, the inclusion of absolute values like lesions/mm^2^ occurred significantly more often in CNS disease scoring than for other organs although this is not comprehensible in each case. Only one scoring system occurred for a peripheral nerve system disease which has been developed to evaluate peripheral nerve ischemia in a relatively simple two-tier system with a zero to four-scale [66].

### Scoring systems for murine models of pulmonary diseases

Fourteen original semiquantitative, multiparametric scoring systems were identified for pulmonary diseases (Table 5). Of these pulmonary fibrosis and pulmonary inflammation were diseases with the highest number scoring systems and citations. For instance, the scoring system developed by Ashcroft, which is a relatively simple multiparametric but single scaled system, is commonly used for the evaluation of lung fibrosis [83]. Three scoring systems were developed for models of general acute lung inflammation (Table 5). They used the parameters of edema and anatomical site specific inflammation as parameters to evaluate the relative amount of inflammatory response. Similar parameters in a wide variety of combinations were used to develop scoring systems for diverse infectious pneumonia models. This variation is again in most cases not based on reasonable argument for the inclusion of a certain parameter in a certain model and therefore not in all cases clearly associated with the supposed pathogen-associated pathogenesis of the respective pneumonia.

**Table 5:** Semiquantitative scoring systems for murine models of pulmonary diseases

**Pulmonary disease models**	**Scoring system: parameters (scale width)**	**Citations**
Lung fibrosis [83]	Alveolar/bronchial wall thickening, structure distortion, fibrosis (together 0–8)	273
Lung fibrosis [84]	Fibroblastic foci, established fibrosis, intraalveolar macrophages (each 0–6)	116
Cystic fibrosis [85]	Lymphoid infiltrate (0–5), goblet cell hyperplasia (0–2), mucus retention (0–3), bronchiolitis (0–5), pneumonia (0–3), edema (0–2)	101
Ventilator-induced lung injury [86]	Alveolar congestion, hemorrhage, neutrophils in airspaces/vessel walls, alveolar wall thickness, hyaline membranes (each 0–4)	78
Pulmonary ischemia [87]	Edema, inflammatory cell infiltration, vascular congestion, alveolar hemorrhage (each 0–3)	5
Smoke-induced pneumopathy [88]	Alveolar emphysema, atelectasis, infiltration, hemorrhage, alveolar wall thickness, perivascular/peribronchiolar edema (each 0–3)	4
Lung inflammation [89]	Perivascular edema (0–3), perivascular/-bronchiolar inflammation (0–3), goblet cell metaplasia (0–2)	32
Lung inflammation [90]	Alvolear wall inflammation, perivenous regions, periarterial/peribronchial regions, venous/arterial endothelial lesion (each 0–3)	30
Acute lung inflammation [91]	Alveolar necrosis, vascular congestion, infiltration by neutrophils/ macrophages (each 0–4)	6
RSV pneumonia [92]	Peribronchiolitis, alveolitis, perivasculitis, hypertrophy of mucus-producing glands, eosinophilia (each 0–5).	26
Mycoplasma pneumonia [93]	Quantity/quality of (peri-)bronchial infiltrates, bronchial luminal exudate, perivascular infiltrate, parenchymal pneumonia (0–3)	56
*Pneumocystis carinii* infection [94]	Cyst number (0–4), inflammation (0–5)	87
Streptococcus pneumonia [95]	Bronchitis, edema, interstitial inflammation, intraalveolar inflammation, pleuritis, endothelialitis (each 0–4)	0

### Scoring systems for myocardial, vascular and muscular disease models

Three original scoring systems for the evaluation of viral myocarditis were identified [96-98]. All of them included the evaluation of the parameters of myocardial necrosis and inflammation (Table 6). In addition, two of them also included calcification as a parameter while fibrosis and Evans blue-staining as a marker of myofiber damage were used as a parameter of myocardial disease only once.

**Table 6:** Semiquantitative scoring systems for cardiovascular and muscle disease models

**Disease model**	**Scoring system: parameters (scale width)**	**Citations**
***Myocardial diseases***		
EMCV-induced myocarditis [96]	Myocardial necrosis, infiltration, calcification, fibrosis (together 0–4)	50
Coxsackievirus-induced myocarditis [97]	Myocardial necrosis, infiltration, calcification (together 0–4)	33
Coxsackievirus-induced myocarditis [98]	Necrosis, inflammation, Evans blue-stain (each 0–4)	17
Dilated cardiomyopathy [99]	Myocardial necrosis, fibrosis (together 0–4)	20
Chronic cardiotoxicity [100]	Qualitative/quantitative myocardial degeneration score (each 0–4)	72
***Vascular diseases***		
Aneurysm [101]	Extent of medial, adventitial disruption/size of lesion (together 0–6)	21
Atherosclerosis [102]	Medial erosion, foam cells, buried fibrous caps, chondrocyte-like cells, lateral xanthomas (each 0,1)	2
Vasculitis [103]	Infiltration, elastic lamina destruction, intimal thickening (together 0–3)	8
***Muscle diseases***		
Ischemic necrosis [104]	Infiltration, necrosis, hemorrhage (together 0–10)	14
Trypanosoma myositis [105]	Number of parasites, eosinophilic infiltration (each 0–3)	0

*EMCV*, Encephalomyocarditis virus.

Three semiquantitative scoring systems for the most important human vascular diseases were identified: atherosclerosis, aneurysms and vasculitis (Table 6) [101-103]. They all cover several aspects of the pathogenesis and pathophysiology of the diseases but have been generally rarely cited yet. The aneurysm scoring system grades the severity of the disease by the extent of medial and adventitial lesion together with the general size of the lesion [102]. The atherosclerosis scoring systems uses a 5-tier system with a 0–1 scale width [102], whereas the vasculitis score uses the parameters infiltration, elastic lamina destruction and intimal thickening, thus indicating that the system may only be useful for evaluation of larger vessel types [103].

Two scoring systems for muscular disease models were identified (Table 6). One recently published scoring system evaluated the extent of *Trypanosoma*-induced myositis [104] while the other scoring system was developed to quantify the extent of ischemia-induced muscle necrosis by the parameters necrosis, infiltration and hemorrhage [105].

### Scoring systems for hepatic and pancreatic diseases

Ten original scoring systems for chronic hepatitis have been developed or used for the quantification chronic hepatic disease (Table 7). The scoring systems by Ishak and Knodell are both highly cited scoring systems and cover almost all possible histomorphologic changes in chronically inflamed livers [106,107]. The two identified scoring systems for acute hepatitis quantify lesions by grading the extent of inflammation and necrosis, similar to the Ishak system for chronic hepatitis [108,109].

**Table 7:** Semiquantitative scoring systems for murine hepatic and pancreatic disease models

**Disease model**	**Scoring system: parameters (scale width)**	**Citations**
***Hepatic disease***		
Chronic active hepatitis [107]	Periportal bridging (0–10), intralobular necrosis, portal inflammation, fibrosis (each 0–4)	2,609
Chronic hepatitis [106]	Periportal/septal inflammation (0–4), confluent necrosis (0–6), focal necrosis/apoptosis/inflammation (0–6), portal inflammation (0–4), fibrosis (0–6)	2,001
Chronic hepatitis [110]	Mitotic activity, portal inflammation, ductular proliferation, councilman bodies, fibrosis (each 0–3)	107
Hepatic fibrosis [108]	Centrolobular vein/perisinusoidal space fibrosis (each 0–2), portal tract fibrosis (0–3), septa number (0–3), septa width (0–5)	135
Acute hepatitis [111]	Steatosis (0–4), necrosis, inflammation (each 0–2),	218
Acute hepatitis [109]	Portal/lobular inflammation (each 0–3)	41
Nutritional hepatopathy [112]	Hepatocyte degeneration, portal inflammation, portal fibrosis (each 0–3)	25
Alcohol-induced hepatopathy [113]	Steatosis (%), inflammation/necrosis/fibrosis (each 0–2)	8
Non-alcoholic Steatohepatitis [114]	Steatosis (0–3), hepatocellular ballooning (0–2), lobular inflammation (0–2)	1,149
Hepatic ischemia [115]	Location, necrosis (together 0–4)	12
***Pancreatic disease***		
Acute pancreatits [116]	Edema, necrosis, inflammation, hemorrhage, fat necrosis (each 0–4)	307
Acute pancreatitis [117]	Edema, necrosis, inflammation, vacuolization (each 0–4)	144
Acute pancreatitis [118]	Edema, necrosis, inflammation, hemorrhage (each 0–4)	89
Acute pancreatitis [119]	Acinar-cell ghosts (%), acinar cells vacuolization/swelling (%)	42
Ischemia-induced acute pancreatitis [120]	Edema, necrosis, infiltration, hemorrhage, vacuolization (each 0–3)	14
Autoimmune pancreatitis [121]	Infiltration, necrosis, lipomatosis (0–4)	42
Insulitis [122]	Islet infiltration, destruction, atrophy (0–5)	49

Five scoring systems for the evaluation of acute pancreatitis have been identified (Table 7). The first and most commonly cited scoring system was published by Schmidt et al. [116]. It uses the five parameters edema, necrosis, inflammation, hemorrhage and fat necrosis to score the extent of pancreatic lesions. All four later developed scoring systems only marginally modified the parameters by omitting a single parameter or including vacuolization as an additional marker (Table 7). In addition, one multiparametric but several uniparametric (data not shown) scoring systems were identified for the quantification of insulitis in mice models. The scoring system by Papaccio et al. uses islet infiltration, atrophy and destruction as parameters for the evaluation of Isle of Langerhans inflammation [122].

### Scoring systems for skin and ocular diseases and miscellaneous disease models

Murine psoriasis models are the only skin disease model with more than one identified scoring system (Table 8). Both scoring systems offer a wide variety of parameters for the evaluation of epidermal and dermal changes in models of this relevant human disease [123,124]. In addition, scoring systems for dermal sclerosis, burn scars, atopic dermatitis and epithelial irritation were identified (Table 8).

**Table 8:** Semiquantitative scoring systems for murine models of eye, skin and miscellaneous diseases

**Disease model**	**Scoring system: parameters (scale width)**	**Citations**
***Skin diseases***		
Burn scars [125]	Epidermal hyperplasia/hyperkeratosis, hair follicles, apocrine glands, smooth muscles, fibroplasia, vascular proliferation (each 0,1), collagen orientation (0–3)	26
Systemic sclerosis [126]	Dermal inflammation, thickened collagen bundles, dermal thickness (each 0–3)	18
Atopic dermatitis [127]	Epidermal hypertrophy, hyperkeratosis, parakeratosis, erosion, inflammation, edema, ulcer (each 0–4)	8
UV radiation-induced skin damage [128]	Epidermal thickness (0–3), dermal cellularity (0–3), dermal cyst changes (0–5)	7
Epithelial irritation [129]	Leukocyte infiltration (0–5), epithelial reaction (0,1)	1
Psoriasis [123]	Munro abscesses (1.5), hyperkeratosis (0.5), parakeratosis (1), length of rete ridges (0.5–1.5), lack of granular layer (1), acanthosis (1), dermis lymphocytic infiltrate (0.5–1.5), papillary papillae congestion (1), thinning above papillae (0.5)	18
Psoriasis [124]	Epidermal thickness, *Stratum corneum* thinning, extent of *Stratum granulosum*/parakeratosis/inflammation, microabscesses (each 0–3)	5
***Ocular diseases***		
Diabetic retinopathy [130]	Inflammation (leukocytes/100 μm retina length), leukostasis (leukocytes per vessel lumen)	25
Oxygen induced retinopathy [131]	Blood vessel growth, tufts, tortuosity, extraretinal neovascularization, vasoconstriction (each 0–3), hemorrhage (0,1)	48
Bacterial endophthalmitis [132]	Inflammation, retinal architecture (each 0–4),	18
Autoimmune uveoretinitis [133]	Infiltration (0–4)	285
Endotoxin uveitis [134]	Inflammation (0–3)	47
***Miscellaneous diseases***		
Abdominal adhesion [135]	Inflammation, fibrosis, necrosis/abscess, granulomas (each 0–3)	100
Abdominal adhesion [136]	Vessel number, neutrophil infiltration, neutrophils at site (each 0–3)	4
Abdominal adhesion [137]	Fibrotic matrix, collagen fibers, fibroblast proliferation (together 1–5)	n.r.
Embryonic development [138]	Grading (0–5) of 17 parameters	429
Wound healing [139]	Infiltration, granulation tissue, fibroblasts, collagen deposition (together 0–12)	369
Thyroiditis [140]	Number of inflammatory foci, parenchymal destruction (together 0–4)	77
Vaginitis [141]	Epithelial disruption, leucocyte infiltration, edema, vascular injection (each 0–4)	74
Esophagitis [142]	Epithelial damage/hemorrhage (0–4), inflammation (0–3)	6
Lymph node [143]	Heterophils, apoptotic histiocytes, sinus histiocytosis, follicular hyperplasia (together 0–5)	1
Spermatogenic activity [144]	Presence of spermatozoa/spermatides/germ cells/sertoli cells (together 0–10)	n.r.

*n.r.*, not reported; *UV*, ultraviolet light.

Five original scoring systems for the evaluation of ocular diseases were identified. Two of these systems use only one parameter for the evaluation of autoimmune and endotoxin uveitis. For the sake of completeness these scoring systems are also displayed in Table 8, although they do not fulfill requirements for inclusion [133,134]. Furthermore, the identified scoring system for diabetic retinopathy uses two parameters evaluated in absolute numbers of leukocytes per area [130].

Three scoring systems for the evaluation of abdominal adhesions after traumatic or toxic irritation of the peritoneum could be identified. Interestingly, not all scoring systems for abdominal adhesion use fibrosis as a parameter for the grading of the adhesions [135-137]. Finally, very helpful scoring systems for the evaluation of embryonic development and wound healing could be identified (Table 8).

### Scoring systems for systemic diseases and transplant rejection

Three original scoring systems analyzing lesions associated with graft-versus-host disease (GvHD) are available (Table 9). The most cited scoring system by Hill et al. exclusively covers intestinal lesions associated with GvHD [145]. This system allows a very thorough analysis of intestinal lesion using a wide variety of parameters in the small and large intestine. The other two systems also cover intestinal lesions but provide additional parameters for the analysis of hepatic [146] or hepatic and skin lesions [147].

**Table 9:** Semiquantitative scoring systems for systemic disease models

**Disease model**	**Scoring system: parameters (scale width)**	**Citations**
***Graft-versus-host disease (GvHD)***		
Intestinal acute GvHD [145]	Small intestine: villous blunting, crypt regeneration/apoptosis/loss, enterocyte loss, infiltration (each 0–4); Colon: crypt regeneration, colonocyte vacuolization, crypt apoptosis/destruction, infiltration (each 0–4)	389
Acute GvHD [146]	Small/large bowel: villous blunting, crypt regeneration/apoptosis/loss, luminal sloughing, infiltration, mucosal ulceration, epithelial/vacuolization (each 0–4), liver: portal infiltration, bile duct apoptosis/sloughing, parenchymal apoptosis/abscesses/mitoses, steatosis, cholestasis (each 0–4)	170
Acute GvHD [147]	Skin: epidermal damage, dermal collagen density, dermal infiltration, subcutaneous fat loss, hair follicle loss (each 0–2), intestine: crypt apoptosis, inflammation(each 0–4), liver: bile duct injury (0–4)	50
***Hemorrhagic shock***		
Lung lesion [148]	Alveolar membrane thickening, congestion, edema, intraalveolar hemorrhage, interstitial, intraalveolar infiltration (each 0–3)	36
Intestinal, pulmonary lesions [149]	Intestine: % injury (number of edematous villi x 0,5 + number of villi with epithelial damage x 1), lung: number of neutrophils in 10 fields	2
Pulmonary, intestinal, renal, hepatic lesions [150]	Lung: atelectasis, hemorrhage, edema, congestion, inflammation, hyp-eraeration (each 0–3); ileal mucosal damage, (0–5), liver: congestion, necrosis, vacuolization (each 0–3), Kidney: epithelial swelling, tubular dilation, necrosis, edema, microthrombosis (each 0–3)	2
***Immunotoxicity***		
[151]	Complete assessment of all lymphoid organs (each 0–4)	50
***Transplant rejection***		
Banff classification for renal rejection [152]	Tubulitis, arteritis, mononuclear cell interstitial infiltrates, glomerulitis, interstitial fibrosis, tubular atrophy, glomerulopathy, mesangial matrix increase, vascular fibrous intimal thickening, arteriolar hyaline thickening (each 0–3)	1.727
Heart rejection 1990 [153]	Infiltration, myocyte damage (0–4)	1401
Heart rejection 2005 [154]	Infiltration, myocyte damage (0–3)	365
Lung rejection [155]	Inflammation (0–4), lymphocyte infiltration (0–1), bronchiolitis obliterans (0–4), Vascular rejection (0–1), vasculitis (0–1)	362
Skin rejection [156]	Acanthosis, ulceration, necrosis, inflammation, granulation tissue (0–5)	1

*GvHD*, Graft versus host disease.

Three original scoring systems have been developed for the analysis lesions associated with hemorrhagic shock (Table 9). The scoring system with the highest citation number only focusses on the pulmonary lesion and offers a variety of parameters for the evaluation of shock-induced lesions in the lung [148]. The other two scoring system also include parameters for lung evaluation but both offer additional parameters for the quantification of intestinal changes [149,150] or in one case offer scoring systems for renal and hepatic lesions [150] associated with hemorrhagic shock.

The identified scoring system for immunotoxicity of toxins more or less demands the analysis of all immune organs but gives a good guideline in terms of the nomenclature of morphologic changes associated with the toxin application (Table 9) [151].

Finally, the three scoring systems which have been used for murine transplant rejection models have consistently been developed for the evaluation of tissues from human patients (Table 9). They are all used in mouse models unmodified to allow for better conclusions from the mouse models for the situation in the human patient.

## Discussion

Extensive research of the literature identified 146 originally designed semiquantitative, multiparametric scoring systems for the histopathology of mouse models. These scoring systems cover almost all organs systems and a wide variety of disease models. Colitis and especially ulcerative colitis was the disease model with the largest number of different scoring system closely followed by experimental autoimmune encephalitis (EAE), lupus nephritis and collagen induced osteoarthritis.

The number of citations for the publication including the scoring system varied between few citations and up to 2176. The citation number clearly reflects the value of the scientific work shown in the papers and thus also indirectly reflects the quality of the included scoring systems. In some cases there is even clear evidence that the high citation number is directly based on the “gold” standard character of the scoring system and its regular use in mouse models or human tissues, for instance the score from Mankin et al. for osteoarthritis evaluation and the scores developed by Knodell and Ishak et al. for chronic hepatitis or the score by Cooper et al. for DSS-colitis [4,28,106,107]. Nevertheless, after careful analysis of the publication it also became obvious that scoring systems in publications with a small number of citation also proofed to be of expedience for certain scientific question.

Assuming that the main function of scoring systems is the analysis of the influences of experimental factors on the microscopical tissue morphology the selected parameters should be consciously chosen to be able to reflect the potential changes. The lack of a rationale for the selection of the parameters was therefore an emerging and surprising finding during the literature search for this study. Although the selection of parameters in most scoring systems is comprehensibly based on the common knowledge on the pathogenesis of the disease modeled in the mouse, there is only rarely a clear statement or a line of argument for choosing a parameter. Even less often the correlation between scoring parameter value and the magnitude of the clinical symptoms or the differences in the extent of the experimental factor is given as for instance in the excellent study of Cooper et al. [4]. This lack is most probably due to the time-consuming work involved, but it may however tremendously increase the value and the scientific merit of the scoring system.

## Conclusion

In summary, a final judgment of the quality and the usefulness of the scoring systems presented was not an aim of this study and is after all most probably not possible since the value of a scoring system clearly depends on the scientific question, the underlying hypothesis, the model characteristics and the pathogenesis of the disease. This review may however give an overview on currently available scoring systems and may therefore allow for a better choice for the respective project.

## Methods

### Selection of scoring systems

The systematic review was prepared according to the PRISMA guidelines [157]. All items were considered and can be viewed in Additional file 1. Scoring systems were identified by a comprehensive Pubmed search (http://www.ncbi.nlm.nih.gov/pubmed/) using a combination of the search terms “mouse”, “score”, “histopathology”. This led to the identification of 1479 publication by October 30, 2012 (Figure 1). Full text versions of all publications were obtained and analyzed for the description of multiparametric, semiquantitative, scoring systems for the histopathology of mouse models. Inclusion of a mouse scoring system in this overview was based on the fulfillment of six parameters.

First, the scoring system had to be based on the semiquantitative evaluation of histopathologic changes in murine tissues. Thus, approaches using digital image analysis for absolute quantification of lesion area, cell number or immunohistochemical signals or scoring systems with dominance of immunohistochemical markers as evaluation parameters were not included.

Second, only scoring systems evaluating more than one histomorphologic parameter were included in the review. Nevertheless, scoring systems with high citation numbers which combined several parameters in a uniparametric score were also included. For instance, if a highly cited scoring system integrated the presence and extent of crypt abscesses, epithelial sloughing and submucosal infiltration into a single score of 0 to 4 the study was also included.

Third, the scoring approach had to be comprehensibly described to allow for reproduction by the reader.

Fourth, the scoring system had to be originally designed for the presented study without citation of former publications. If former publications were cited as the source of the scoring system, the string of citations was followed back to the study originally describing the scoring systems. If scorings systems were not referenced to older studies but similar approaches were detected in earlier publication, only the older study was included in this review.

Fifth, the scoring systems were generally grouped by the organ affected and analyzed. Systemic diseases and transplantation models were included in separate groups. If the number of identified scoring systems for a specific disease model exceeded ten, only the then most cited scoring systems were included in this review.

Citation number was obtained using Thomson Reuters Web Science^©^ (http://apps.webofknowledge.com).

## Competing interests

The author declares that he has no competing interests.

## Author’s contributions

RK had the idea of the project, performed the internet search, the data analysis and wrote the abstract.

## Supplementary Material

Additional file 1 PRISMA 2009 Checklist. Click here for file
